# Coats disease with retinal neovascularization under subfoveal nodule: optical coherence tomography-angiography findings before and after ranibizumab treatment

**DOI:** 10.5935/0004-2749.20210062

**Published:** 2021

**Authors:** Sema Oruc Dundar, Furkan Verdi, Ayse Ipek Akyuz Unsal

**Affiliations:** 1 Aydin Adnan Menderes University, Faculty of Medicine, Department of Ophthalmology, Aydin, Turkiye

**Keywords:** Retinal telangiectasis, Retinal neovascularization, Fluorescein angiography/methods, Tomography, optical coherence, Fovea centralis, Ranibizumab/therapeutic use, Laser coagulation, Telangiectasia retiniana, Neovascularização re tiniana, Angiofluoresceinografia/métodos, Tomografia de coer ência óptica, Fóvea central, Ranibizumab/uso terapêutico, Fotocoagulação a laser

## Abstract

We conducted retinal neovascularization under subfoveal fibrotic nodule for Coats disease by using optic coherence tomography-angiography before and after ranibizumab treatment. Our patient was an 8-year-old boy who was referred with suspicious left retinal mass. His visual acuity was 20/400 in the left eye and 20/20 in the right eye at the time of admission. Posterior segment evaluation of the left eye revealed telengiectatic vessels at the inferotemporal region of the peripheral retina with hard exudates around the optic disc and macula typical for Coats disease. His optic coherence tomography revealed a subfoveal fibrotic nodule after ranibizumab injections and laser photocoagulation treatment. The optic coherence tomography-angiography results revealed neovascularization under the subfoveal nodule at the superficial vascular complex layer. After 3 intravitreal ranibizumab injections, his neovascularization regressed on optic coherence tomography-angiography and his visual acuity improved. To the best of our knowledge, this is the first report demonstrating neovascularization under the subfoveal fibrotic nodule in Coats disease on the basis of comparative with the help of optic coherence tomography-angiography before and after the treatment.

## Abstract (pt)

Demonstramos uma neovascularização da retina sob o nódulo fibrótico subfoveal na doença de Coats com a ajuda da Angiotomografia de Coerência Óptica (OCT-A) antes e após o tratamento com ranibizumabe. Paciente do sexo masculino de 8 anos foi encaminhado com suspeita de massa retiniana no olho esquerdo. A acuidade visual foi de 20/400 no olho esquerdo e de 20/20 no olho direito. A avaliação do segmento posterior do olho esquerdo revelou vasos telengiectáticos na região inferotemporal da retina periférica e exsudados duros em torno do disco óptico e mácula típica da doença de Coats. A angiotomografia de coerência óptica apresentou nódulo fibrótico subfoveal após injeções de ranibizumabe e tratamento com fotocoagulação a laser. A angiotomografia de coerência óptica mostrou neovascularização sob o nódulo subfoveal na camada superficial do complexo vascular. Após três injeções de ranibizumabe intravítreo, a neovascularização regrediu na angiografia por tomografia de coerência óptica e a acuidade visual melhorou. onde sabemos, este é o primeiro relato a mostrar neovascularização sob nódulo fibrótico subfoveal na Doença de Coats com a ajuda da angiografia por tomografia de coerência óptica antes e após o tratamento.

## INTRODUCTION

Since the day Coats^(1)^ described a retinal vascular abnormality with unilateral exudation in the retina of young men, new diagnostic tools and treatment options have emerged for the treatment of this idiopathic exudative retinopathy^(2-4)^. After Shields’ classification^(5)^, Daruich et al.^(6)^ defined subfoveal nodule as a herniated exudative subfoveal yellow spheroidal lesion. Several papers have illustrated subfoveal nodule and macular exudates or scars in association with vascular components, but none demonstrated the early phase of subfoveal nodule with a neovascularization at the superficial vascular layer^(7-10)^. We have presented an extraordinary retinal neovascularization under subfoveal fibrotic nodule by performing comparative observations with optical coherence tomography-angiography (OCT-A) before and after treatment with intravitreal (i.v) anti-vascular endothelial growth factor (anti-VEGF) injections.

## CASE REPORT

**Figure 1:**
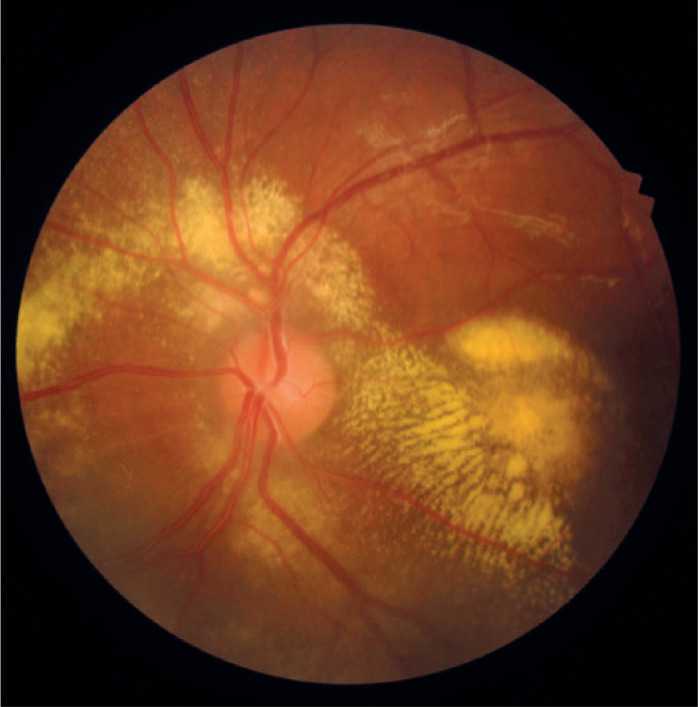
Initial fundus photo of the left eye: hard exudates can be seen around the optic disc and macula.

**Figure 2:**
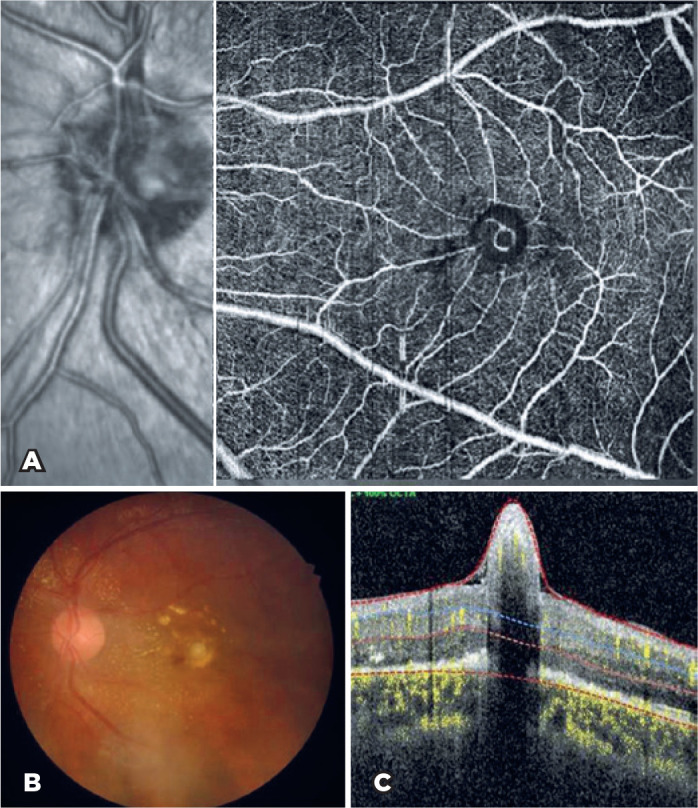
A) OCT-A depicting neovascularization at the superficial vascular complex layer under the herniated subfoveal nodule. B) Intraretinal hemorrhage nasal to fovea and subfoveal nodule can be seen on the fundus photo. C) Herniated subfoveal fibrotic nodule with a shadowing effect under the fovea can be seen on OCT.

**Figure 3:**
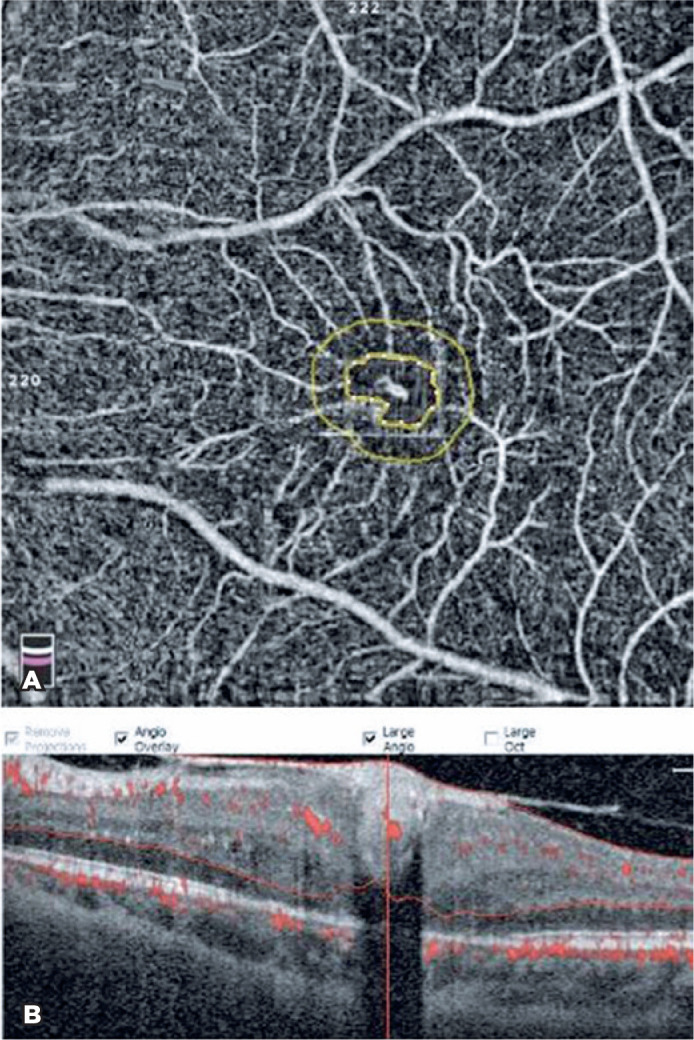
A) OCT-A showing the shrinkage of retinal neovascularization after intravitreal ranibizumab injection to the superficial vascular layer. B) The regression of subfoveal fibrotic nodule demonstrated on OCT.

An 8-year-old boy was referred to our clinic with suspicious left retinal mass. His visual acuity was 20/400 in the left eye and 20/20 in the right eye. His anterior segment examination and magnetic resonance imaging of the orbit showed unremarkable results. The fundus of the right eye was normal. The retinal telengiectatic vessels at the inferotemporal region and hard exudates around the optic disc and macula indicated Stage 2B Coats disease (Figure 1). After 2 sessions of panretinal laser photocoagulation (PRP), subfoveal fluid was observed on OCT and i.v ranibizumab injection was administered in combination with laser photocoagulation. After administering 3 injections, intraretinal hemorrhage nasal to the fovea and a herniated subfoveal fibrotic nodule were detected on funduscopic examination. The patient’s OCT findings revealed an elevated hyper-reflective lesion with a shadowing effect under the fovea. Intraretinal hyper-reflective dots, enlargement of the foveal avascular zone (FAZ), and the epiretinal membrane were also observed. OCT-A revealed neovascularization at the su perficial vascular complex layer (Figure 2) despite the absence of neovascularization in the deep vascular plexus. The patient accordingly underwent an additional 3 i.v. ranibizumab injections. After the treatment, fibrotic nodule reduced and retinal neovascularization at the superficial vascular layer shrunk on OCT-A (Figure 3). The visual acuity increased to up to 20/100.

## DISCUSSION

Khurana et al.^(10)^ was the first to report subfoveal nodule as an atypical initial presentation of Coats disea se. Since then, Daruich et al.^(6)^ defined it as a yellow, protruding, spheroidal exudative lesion and proposed a subcategory for Stage 2B, including subfoveal nodule as Stage 2B2. The size and shape of the subfoveal nodule that we have presented here are smaller, thinner, and lacking retinal-retinal anastomosis, hyperpigmentation, associated parafoveal nodule, and secondor third-order retinal vessel entering the nodule, different from those in the other cases in the literature^(6-8)^.

Rabiolo et al.^(2)^ demonstrated that 75% of type 3 neovascularization in patients with macular fibrosis originate from the choroid and retina and are associated with FAZ replacement and their absence; it is possible that the neovascularization in our case originated from the choroid similar to these. However, we could not confirm the same with indocyanine green angiography our patient. In addition, the FAZ of our patient was enlarged, unlike in these earlier cases.

Hautz et al.^(8)^ evaluated a group of patients with OCT-A and demonstrated aneurysm-like dilations and vascular loops on superficial capillary plexus of the macular region in Coats disease. Although the authors found that the macular scars have intralesional vessels, they could not confirm whether these vessels were a result of neovascularization. In our case, OCT-A revealed neovascularization under the subfoveal nodule at the superficial vascular complex layer. Shrinkage of the vessel after ranibizumab injections also confirmed our notion that the vessel was indeed a result of neovascularization.

Schwartz et al.^(7)^ compared the vascular density and FAZ in 7 eyes with Stage 2B Coats disease versus 7 unaffected fellow eyes. They found that the vascular density decreased and the FAZ enlarged, especially at Stage 2A. The enlarged FAZ in our case was also consistent with that in the aforementioned study, and the early demonstration of neovascularization on OCTA compelled us to initiate the treatment earlier with anti-VEGF. The intraretinal hemorrhage nasal to the fovea supports the idea that a vessel at the superficial vascular layer may be the point of retinal neovascularization in our case. However, this intraretinal hemorrhage could have obscured the retinal-retinal or chorioretinal anastomosis of the neovascularization in the superficial vascular complex layer.

The present case report illustrates the benefit of applying anti-VEGF treatment for resolving subfoveal nodule associated with a vascular component before the initiation of macular fibrosis. As OCTA is a non-invasive and rapid tool to demonstrate vascular alterations in pediatric patients, it can be useful to demonstrate the vascular component of subfoveal nodule earlier so as to accordingly initiate the treatment strategy and the visual outcomes.

In conclusion, to the best of our knowledge, this is the first report demonstrating the application of OCT and OCT-A images in the treatment of subfoveal nodule formation with neovascularization that resulted in the improvement of the visual acuity after anti-VEGF treatment.
